# Importance of the Lunar Cycle on Mesopelagic Foraging by Atlantic Bluefin Tuna in the Upwelling Area of the Strait of Messina (Central Mediterranean Sea)

**DOI:** 10.3390/ani12172261

**Published:** 2022-08-31

**Authors:** Pietro Battaglia, Cristina Pedà, Danilo Malara, Giacomo Milisenda, Brian R. MacKenzie, Valentina Esposito, Pierpaolo Consoli, Teresa Manuela Vicchio, Maria Giulia Stipa, Luca Pagano, Francesco Longo, Teresa Romeo

**Affiliations:** 1 Stazione Zoologica Anton Dohrn, Sicily Marine Centre, Villa Pace, Contrada Porticatello n. 29, 98167 Messina, Italy; 2 Stazione Zoologica Anton Dohrn, Calabria Marine Centre, CRIMAC, Integrated Marine Ecology Department, C. da Torre Spaccata, 87071 Amendolara, Italy; 3 Stazione Zoologica Anton Dohrn, Sicily Marine Centre, Ex Complesso Roosevelt, Lungomare Cristoforo Colombo, 4521 Palermo, Italy; 4 National Institute of Aquatic Resources (DTU Aqua), Technical University of Denmark, DK 2800 Kongens Lyngby, Denmark; 5 OGS (Istituto Nazionale di Oceanografia e di Geofisica Sperimentale), 34014 Trieste, Italy; 6 Stazione Zoologica Anton Dohrn-Integrative Marine Ecology, Sicily Marine Centre, Via dei Mille 46, 98057 Milazzo, Italy; 7 Istituto Superiore per la Protezione e la Ricerca Ambientale (ISPRA), Via dei Mille 46, 98057 Milazzo, Italy

**Keywords:** midwater food resources, mesopelagic prey, lunar irradiance, currents, food web, moon phases, *Thunnus thynnus*

## Abstract

**Simple Summary:**

We investigated the influence of the lunar cycle on bluefin tuna prey composition in the Strait of Messina by stomach content analysis. We tested if the lunar phases could determine changes in mesopelagic prey composition and abundance. Moreover, we considered two potential impacts of the lunar cycle: the lunar irradiance and the strength of currents. These factors could affect availability of mesopelagic prey in upper waters of the study area. Mesopelagic fish and cephalopod prey were 60.7% of overall diet by number. In summary, the Strait of Messina has highly specific hydrodynamic and biological features which strongly depend on upwelling currents, which in turn are influenced by the lunar cycle (new and full moon with strong currents, quarters with fewer currents). Upwelling causes water mixing, bringing to the surface a large amount of mesopelagic fauna which become more readily available to tuna. Lunar irradiance contributes to the variation of prey composition, increasing the success of visual predation on mesopelagic resources at high light in the water column.

**Abstract:**

The influence of the lunar cycle on bluefin tuna foraging in the upwelling area of the Strait of Messina was investigated by exploring trophic interaction with mesopelagic fish and cephalopod prey. To focus on how the lunar cycle could affect availability of mesopelagic prey for this predator, we tested potential differences in the diet related to each lunar phase. Moreover, we considered two potential impacts of the lunar cycle: the lunar irradiance and the strength of currents. Overall, 2672 prey items were mesopelagic fish and cephalopods, representing 60.7% of overall diet by number. The main mesopelagic fish prey items were lanternfishes and dragonfishes, while *Onychoteuthis banksii* was the most important cephalopod prey. In summary, the Strait of Messina has highly specific hydrodynamic and biological features which strongly depend on upwelling currents, which in turn are influenced by the lunar cycle (new and full moon with strong currents, quarters with fewer currents). Upwelling causes water mixing, bringing to the surface a large amount of mesopelagic fauna which become more readily available to tuna. Lunar irradiance contributes to the variation of prey composition, increasing the success of visual predation on mesopelagic resources at high light in the water column.

## 1. Introduction

The lunar cycle has important effects on the marine environment, driving tidal currents, regulating light intensity during nighttime and influencing the ecology and reproductive cycle of several marine organisms [1,2,3]. In the open ocean, lunar irradiance is a limiting factor for the extent of micronekton diel vertical migration in the water column [4,5,6]. According to Prihartato et al. [6], the lunar cycle significantly affects the micronekton distribution on a global scale and the brightest moon phase (full moon) causes a deepening of the deep scattering layer (DSL), although these authors did not detect significant differences in the DSL vertical distribution between new moon and intermediate moon phases. As hypothesized in several studies, the deepening of DSL during the brightest phase of the lunar cycle seems related to a defensive behavior by micronekton to avoid predators [4,5,6,7,8]. Most mesopelagic species carry out nycthemeral migrations toward the epipelagic zone during the night to feed on their prey and move back to deeper waters during daytime [9]. However, each species occupies a particular trophic niche and displays different vertical distribution, adaptations, migratory and feeding habits [10,11]. The water column illumination influences the vertical distribution and migratory behavior of each mesopelagic species, affecting their vulnerability to predation by visually foraging predators in the upper water layers. For this reason, mesopelagic species tend to follow a preferred light intensity (or isolume) in the water column in order to maintain camouflage and hide themselves from predators; avoidance of predators is also assisted by activation of bioluminescent counterillumination strategies [12,13,14,15]. Interspecific differences in the amplitude of vertical distribution of mesopelagic micronekton at night [16] are then also related to the ability of each species to address camouflage and perform defensive tactics [4,11,17]. 

On the other hand, the foraging of visual predators is also influenced by the lunar cycle: predators that require high environmental light levels to encounter and catch prey may limit their foraging activity to the more highly illuminated upper waters [18]. Large pelagic fish such as swordfish and tunas are able to forage on mesopelagic fauna, thanks to their ability to perform depth excursions as shown by satellite tagging studies [19,20,21,22] or dietary studies [23,24,25]. However, the extent of these excursions differs by species, and, according to current knowledge, the deepest dives are found in *Xiphias gladius* and *Thunnus obesus*, which probably have a more specialized visual system for dark environment (e.g., [26]). Recently, some authors observed high percentages of mesopelagic fish and cephalopod prey in the stomach content of Atlantic bluefin tuna (BFT), *Thunnus thynnus*, in different foraging areas of the Atlantic Ocean [27] and the Mediterranean Sea [23,25,28,29]; according to these studies, as well as tagging experiments [19,20], BFT is able to perform vertical excursions to match its food resources and feed on mesopelagic prey. The chance to encounter mesopelagic prey might increase during nighttime, when these species perform nycthemeral migrations (from deep waters toward upper layers).

Furthermore, recent studies demonstrated that, in particular areas, the lunar cycle can also influence the abundance of mesopelagic resources and their availability for predators. This can happen in areas with specific configurations of bathymetry and ocean-climate conditions whose interactions can affect the intensity of tidal currents and the amplitude of upwelling and mixing phenomena [10,30,31]. One such area is the Strait of Messina (central Mediterranean Sea), where the lunar cycle plays an important role in regulating the transport of deep fauna toward the surface layers by modulating the intensity of currents [10,30]. These currents are stronger during full moon and new moon phases and weaker during quarters, and frequently result in the stranding of mesopelagic fauna on local beaches [30]. These biological and physical features provide foraging opportunities for predators and make the Strait of Messina a preferential foraging area for several pelagic predators, including tunas [23,25], billfishes [23,32] and carangids [10,33]. In particular, the Strait of Messina is an important feeding ground for *T. thynnus* during the prespawning and spawning periods, i.e., from late winter to the end of spring [25].

### 1.1. Upwelling System in the Strait of Messina: Hydrodynamic Conditions and Biological Features

**Figure 1:**
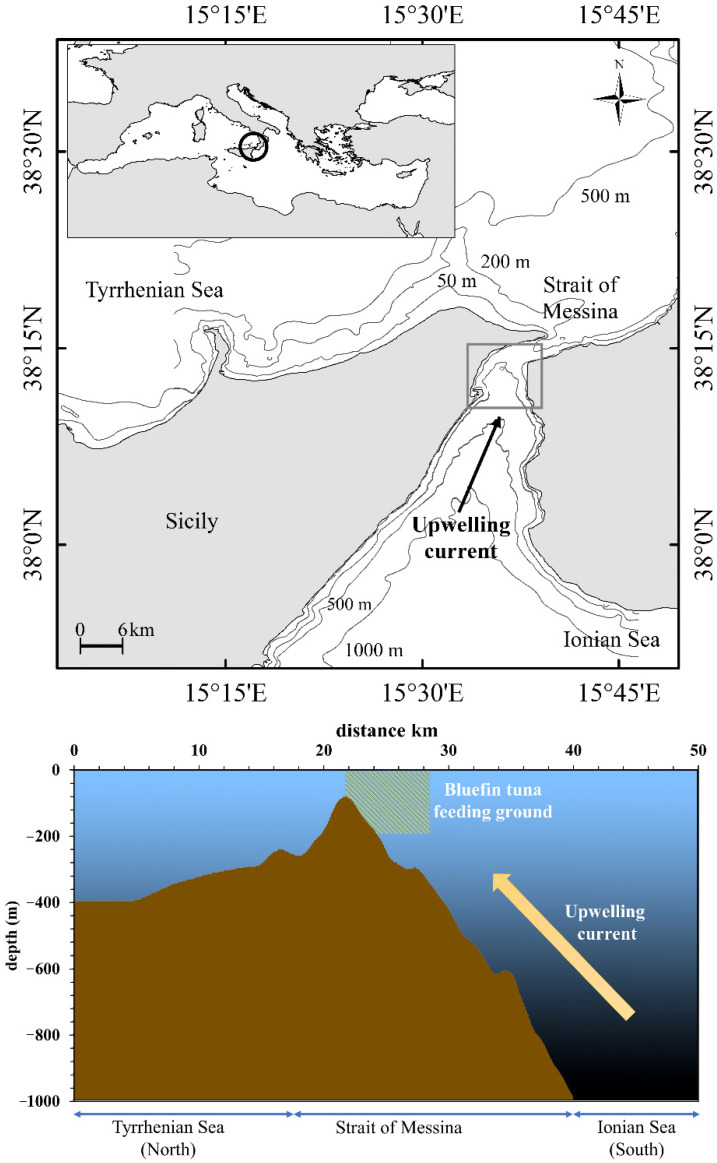
Study area located in the Strait of Messina (central Mediterranean Sea), depth profile of the bottom and direction of the upwelling current. The location of the bluefin tuna feeding ground is also shown.

The Strait of Messina (Figure 1) is a narrow waterway that connects the Ionian and Tyrrhenian Seas and is an important upwelling area of the central Mediterranean Sea. The geographical conformation (relatively wide, ca. 30 km, in the south, narrowing to ca. 3.3 km in the north), bottom profile (rising from about 1500 m in the southern part to 80 m in the northern part; Figure 1) and the difference in water density between Ionian and Tyrrhenian Seas are the main reasons of the peculiar hydrodynamic regime of this area [31,34,35,36,37]. The local hydrodynamic processes are regulated by semidiurnal inversions of the tidal phase between the two interconnected wider basins: when high tide occurs in the Ionian Sea, the low tide occurs in the Tyrrhenian Sea, and vice versa, determining a difference in water level and a subsequent alternate forcing of large currents across the Strait of Messina [34]. Density currents, drift currents, upwelling of waters and other wind-induced perturbations complete the hydrodynamic regime of this area [34,38]. Here, the lunar cycle influences the intensity of tidal currents, which approximately reach maximum speed during new and full moon phases [35]. A result of this hydrodynamic regime is the upwelling of colder, saltier and nutrient-rich Ionian deeper water, which flows northward [31,36,37,39,40], providing a periodic input of nutrients and sustaining primary production [31]. According to Fortier [41], this produces rapid trophic effects on the food web, maintaining the zooplankton and micronekton communities and assuring a high biodiversity level [10,30,42].

### 1.2. Aim of This Study

In this paper, we assess how the lunar cycle can influence the foraging activity of Atlantic bluefin tuna (*Thunnus thynnus* L.) on mesopelagic fish and cephalopod prey in the upwelling area of the Strait of Messina (central Mediterranean). The abundance of mesopelagic fauna in the Strait of Messina, thanks to upwelling currents, has significant ecological implications in the trophic web, representing an important source of food for the bluefin tuna. We used stomach content data collected in the past for the purpose of describing the diet of this large predator in the study area (a general description of the diet of *T. thynnus* has been provided in the related paper [25]). Here, we have further investigated some of these data, focusing our attention on important mesopelagic prey (fish and cephalopods) and considering the lunar cycle as one of the most important factors in the regulation of hydrodynamic regimes and biotic features in the study area. We used this previously collected dietary database, with the addition of some limited new samples, to further explore the trophic interaction between mesopelagic fish and cephalopod prey and BFT, and in particular how the different lunar phases affect availability of these preys to the predator. Furthermore, given that the lunar cycle has a direct effect on the light intensity at night (lunar irradiance) and on the strength of currents, we decided to explore whether these factors may affect the BFT prey composition. It is known that the oscillation of the tidal level is the main process responsible for the upwelling [31]; then, it is assumed that the strength of tidal currents can increase upwelling phenomena in the study area. We hypothesize that the lunar cycle regulates the abundance of mesopelagic prey in upper water layers by influencing the intensity of upwelling currents. We propose that, when the currents become stronger, BFT increases its foraging activity on mesopelagic food resources. We have also evaluated whether the lunar irradiance may affect BFT prey composition, modulating the water column illumination, which influences the vertical distributions and migratory behavior of the mesopelagic fish and cephalopod food resources as well as the vulnerability of these species to visual predation by BFT. 

## 2. Materials and Methods

Our dataset of dietary data consists of prey found in 128 BFT stomachs, collected during late winter (a few specimens caught between the end of February and March) and spring seasons in 2010 and 2011 in the Strait of Messina (central Mediterranean Sea, Figure 1). Samples were obtained at landings from a traditional hand line fishery [25,43,44,45], carried out during daylight by small crafts in the study area. The specimens were caught by hand line and measured (fork length, FL in cm) when landed. Tunas ranged from 110 to 222 cm FL (mean FL = 153.2 ± 27.2 cm). Details of sampling procedures and prey identification are given in Battaglia et al. [25]. Information on lunar phase (new moon, waxing crescent, first quarter, waxing gibbous, full moon, waning gibbous, third quarter, waning crescent) was assigned to each collected sample. Because of the short time scale of lunar phases (on the order of days) and the potential for hard parts to persist in predator guts for longer time scales, we established a cutoff in degree of digestion, excluding prey completely digested, i.e., when only hard parts without flesh remains were found [25,46,47]. Mesopelagic prey items were counted (each individual prey) and measured to the lowest 0.1 mm, recording the standard length (SL) for fish and mantle length (ML) for cephalopods. To avoid overestimation, partially digested prey individuals were counted considering hard structures (otoliths, beaks, vertebral columns, heads, carapaces, etc.). When a partially digested prey was found, individual size was determined using the relationships available in the literature, which allow one to estimate the length of each specimen from fish otolith [48,49] or cephalopod beak [23,50,51,52,53,54] proportions. For this reason, each lower beak was measured to the nearest 0.1 mm, recording the lower rostral length (LRL) for Oegopsida, and the lower hood length (LHL) for Sepiolida and Octopoda. Pelagic octopuses of Argonautoidea were included in mesopelagic prey because, according to Jereb et al. [55], their depth range varies between epipelagic and upper mesopelagic waters. Otoliths (sagittae) were measured to the nearest 0.1 mm. Sagittal length was measured in parallel to the sulcus as the longest distance between the sagitta anterior tip and posterior edge [48,49].

For each mesopelagic fish and cephalopod prey resource, the abundance percentage in the stomach (*%N* = number of individuals of prey *i*/total number of preys) and frequency of occurrence (*%F* = number of stomachs containing prey *i*/total number of stomachs containing prey) were calculated [56,57]. In order to assess the importance of mesopelagic prey in the diet, we also provided abundance and occurrence values for other nonmesopelagic prey items, grouping them in other invertebrates and other fish.

In order to investigate how the prey composition varies in relation to the lunar cycle and its importance for the occurrence of mesopelagic fish and cephalopods in BFT foraging, the relative percentage abundance (*%A*), in terms of number of food items, was calculated for each prey group (mesopelagic cephalopods, other invertebrates, mesopelagic fish, other fish) and moon phase:

%*A* = *N*_im_/*N*_m_ 100

where N_im_ is the number of prey macrocategories i during a specific moon phase (m), and N_m_ is the total number of preys during the moon phase m. The mean number of preys ingested by BFT was also calculated for each moon phase. 

The main hypothesis is that the lunar cycle may influence the prey composition of BFT in the study area. Indeed, it is known that moon phases determine periodical changes in the prey assemblage, particularly as regards mesopelagic component of pelagic fauna [30]. In order to test this hypothesis, we explored if the trophic interaction between BFT and its mesopelagic fish and cephalopod prey was affected by the lunar cycle, analyzing the potential differences between prey groups (prey composition) among lunar phases.

Moreover, given that the lunar cycle directly influences both the light intensity at night (lunar irradiance) and current strength (tidal currents and upwelling phenomena in the Strait of Messina), two other hypotheses were also tested, separately:

(1) Differences in lunar irradiance influence the water column illumination and therefore could affect both the predatory activity (visual predation may increase when light is more intense) and the vertical distributions and migratory behavior of mesopelagic fish and cephalopods and, consequently, their vulnerability to BFT predation. Under this hypothesis, we would expect that occurrence of mesopelagic prey in BFT stomachs would be higher with low and high illumination (new moon and full moon, respectively): (i) on one hand, when lunar irradiance is low, the mesopelagic prey would carry out larger vertical migrations and would be more available to the predator; (ii) on the other hand, when lunar irradiance is higher, the visual predation is more efficient and may be used in a larger extent of the water column. The percentage of lunar face illumination was used as proxy of lunar irradiance. The lunar surface reflects light back when sun directly illuminates the moon’s Earth-side face, and the percentage of lunar surface illuminated by sunlight varies across the lunar cycle, with values ranging from 100% (full moon) to 0% (new moon) and reaching 50% in both quarters (first and third quarters). The values of lunar face illumination were retrieved by the website www.eurometeo.com (accessed on 15 January 2019) and associated to sampling days and BFT stomach samples. Within the “lunar irradiance” factor, we considered the following levels: low (0.0–33.3%), moderate (33.4–66.6%) and high (66.7–100.0%).
(2) The influence of lunar cycle on the amplitude of upwelling phenomena in the Strait of Messina probably affects the occurrence of mesopelagic fauna in upper waters, contributing to foraging success of BFT on these prey items. The lunar cycle regulates the strength of tidal currents and consequently increases the upwelling phenomena in the study area, as previously described [10,30,31]. Therefore, we hypothesize that when currents get stronger, i.e., in periods of lunar cycle near or coinciding with full and new moon phases, the availability of prey for BFT increases, thanks to the intensification of the upwelling phenomena: the strong currents generated would passively transport mesopelagic fauna higher in the water column, making them more available to predators foraging at shallower depths in the water column. On the contrary, during lunar phases characterized by slowest currents (first and third quarters), the BFT foraging activity on mesopelagic food resources may be reduced because less prey is pushed toward upper water layers. For these reasons, in order to test this hypothesis, according to current strength’s values recorded during sampling activities, we used three current strength’s categories (weak: 2.00–2.99 knots; intermediate: 3.00–3.99 knots; strong: 4.00–4.99 knots). According to tide tables, we used the daily maximum values of the current flowing from south to north, in the direction which allows the upwelling phenomena. In general, the strongest currents occur during full and new moon, and the slowest currents occur during quarters.

In order to perform these analyses, mesopelagic fish and cephalopod prey items were grouped in seven categories: sepiolids, muscular squids, buoyant squids, pelagic octopods, dragonfishes, lanternfishes and other mesopelagic fish. The prey composition in BFT stomachs was analyzed by the factors “lunar phase” (made up of 8 levels: new moon, waxing crescent, first quarter, waxing gibbous, full moon, waning gibbous, third quarter, waning crescent), “lunar irradiance” (made up of 3 levels: low, moderate, high) and by factor “current strength” (made up of 3 levels: strong, intermediate, weak), separately. 

A multivariate analysis was performed to separately investigate the effect of the “lunar phase”, “lunar irradiance” and “current strength”. Particularly, the relation between predictor variables and prey stomach composition were investigated with partial redundancy analysis (pRDA). We transformed the stomach composition matrix data in Hellinger distance, as it has been shown to be more appropriate for data containing many zeros [58]. The other variables were included as predictors. Based on a Monte Carlo permutation with 999 iterations, the RDA was used with forward selection to filter the relative importance of the explanatory variable. Statistical significance was assessed by comparing the initial F-statistic to the distribution of F-values obtained after 1000 permutations of the response matrix and the goodness-of-fit evaluated with the adjusted R^2^. 

The analyses were completed using the “vegan” package within the open source software R and R-studio [59,60]. 

SIMPER analysis (“vegan” package, [61]) was used to detect the prey categories that contributed most to the average dissimilarity among the different levels of each variable. 

Significant variations in the number of preys in the stomach contents, between different moon phases, were analyzed through generalized linear models. The length of tuna individuals was also included in the model structure as an “offset” so that the effect of tuna size on their ability to prey could also be considered. The linear model was fit through the R package “glmmTMB,” using a “ziGAMMA” error family, because our response variable (number of preys in the stomach), could only take positive values, and also had a high number of zeros. Model checking was conducted through the presence of a normal distribution of the residuals and the absence of any particular pattern of residual distribution. 

Significance of “Moon phases” was tested with likelihood ratio chi-squared tests, conducted with the Anova functions present in the “car” package [62]. Estimated group means, confidence intervals and pairwise comparisons were derived with the “multcomp” package [63].

Finally, in order to provide additional data on the size of mesopelagic prey, the length frequency distribution (SL for fish and ML for cephalopods) was calculated for the most important mesopelagic food items. The relationship between BFT fork length and mesopelagic prey lengths was analyzed using linear regression, and then we estimated the percentage ratio in order to understand how the size of prey affects their vulnerability to tuna. Then data were log_10_ transformed and plotted in histogram figures. Mean, standard deviation (SD) and coefficient of variation (CV) were also calculated for percent ratio values of both mesopelagic fish and cephalopod prey. The total length of cephalopods would be the most appropriate measure to assess the vulnerability of these preys to BFT, but it is not possible, based on current knowledge, to estimate this size metric for all the species in this study. As an alternative, we used the mantle length (ML) as a proxy indicator of cephalopod total length.

## 3. Results

Among all 128 BFT stomachs, only 12 out of 128 (9.4%) were found empty. Overall, 4401 prey were found in the BFT stomach contents, and 2672 of them were mesopelagic fish and cephalopods, representing 60.7% of total food by numbers. Within mesopelagic fish, Myctophidae and Stomiidae were the most abundant, and the main prey species were the lanternfish *Hygophum benoiti* (%N = 15.0; %F = 37.9), *Ceratoscopelus maderensis* (%N = 9.7; %F = 37.9), *Myctophum punctatum* (%N = 3.4; %F = 26.7) and the stomiid *Chauliodus sloani* (%N = 10.1; %F = 21.6), respectively. On the other hand, *Onychoteuthis banksii* (%N = 3.2; %F = 11.2) was the most important cephalopod prey, followed by *Illex coindetii* (%N = 2.6; %F = 36.2) and *Abralia veranyi* (%N = 2.1; %F = 6.9). The majority of cephalopod food items belonged to Ommastrephidae and Onychoteuthidae families (Table 1). Table 1 also associates each prey with the lunar phases in which this prey was ingested. 

**Figure 2:**
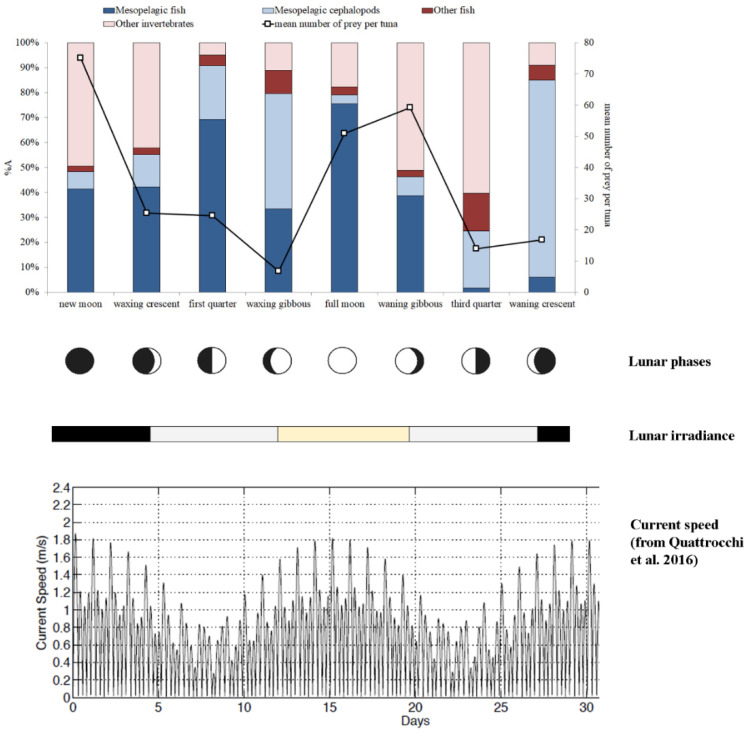
Relative percentage abundance (%A) of food categories consumed by *Thunnus thynnus* during the different moon phases (bar plots). The mean number of preys per tuna across the lunar cycle is also present (line). The lunar irradiance and current strength are also shown for the entire lunar cycle. Lunar irradiance: black = low intensity; grey = medium intensity; yellow = high intensity. Shown as well is a time series of current strength observed during an entire lunar cycle in the Strait of Messina (graph modified from Quattrocchi et al. [64]).

**Figure 3:**
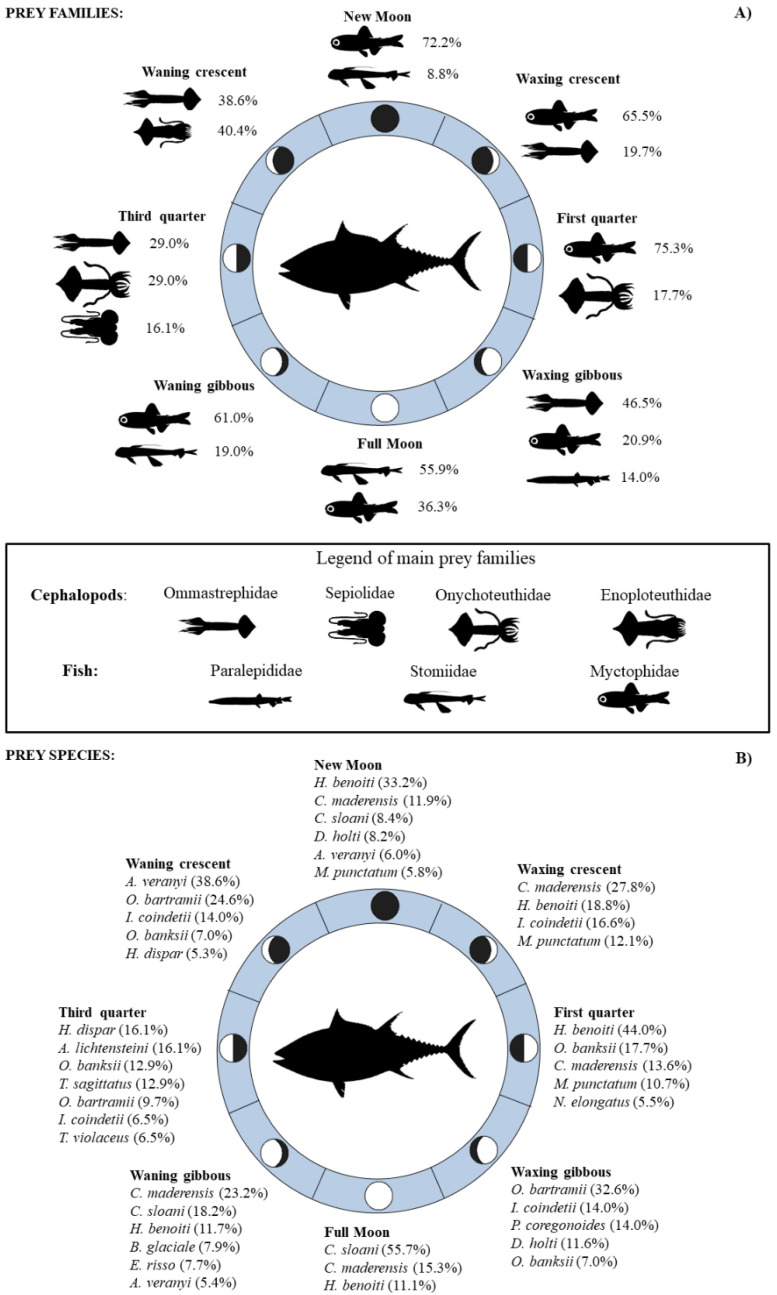
Abundance (%N) of the most important mesopelagic fish and cephalopod prey families (**A**) and species (**B**) consumed by BFT during the different moon phases in the Strait of Messina.

The overall prey group composition varied during the lunar cycle (Figure 2), and differences in BFT mesopelagic prey composition were observed for each moon phase (Figure 3). The mean number of BFT prey was higher during the new moon, full moon and waning gibbous, whereas the lowest values were observed during the waxing gibbous and the third quarter (Figure 2). The relative percentage abundance of mesopelagic fish prey was higher during the full moon (75.5%) and first quarter (69.3%), attaining values between 33.3% and 42.2% during the new moon, waxing crescent, waxing gibbous and waning gibbous phases. In contrast, low values of%*A* for this food category were recorded during the third quarter (1.6%) and the waning crescent (6.0%) moon phases (Figure 2). The values of relative percentage abundance of mesopelagic cephalopods were higher during the waning crescent and the waxing gibbous, at 79.1% and 46.3% of total prey, respectively, although the number of food items ingested during these moon phases was low (Figure 2). 

**Figure 4:**
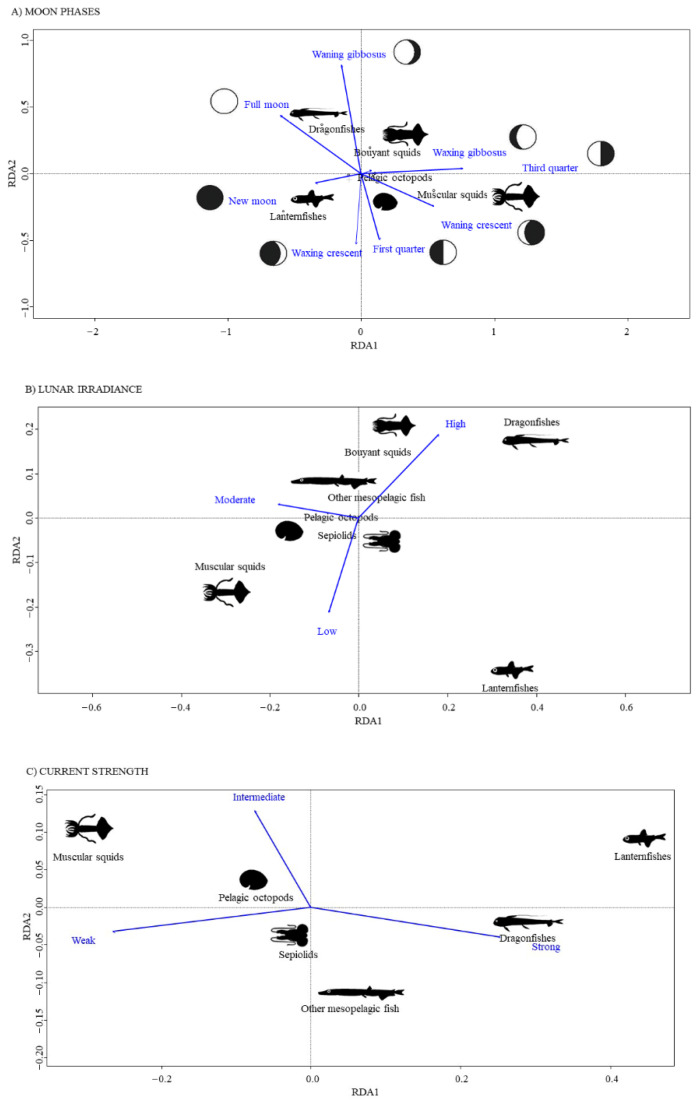
Results of the redundance analysis (RDA) performed on prey composition across lunar phases (**A**) as well as for the factors “lunar irradiance” (**B**) and “current strength” (**C**).

Redundance analysis performed on stomach content composition and lunar phases explained 15% of the total variance in prey composition (*p* < 0.001). The analysis showed a positive correlation mainly between dragonfishes and the “full moon” phase, between lanternfishes and the “new moon” phase and finally between muscular squids and the “waning crescent” phase (Figure 4A).

Lunar irradiances explained only 7.3% of total variance in stomach prey composition (*p* < 0.01). Dragonfishes and buoyant squids were most responsible for the difference in prey composition of BFT stomachs when lunar irradiance was high, while muscular squids and lanternfishes mostly contributed at medium and low light intensities, respectively (Figure 4B).

The intensity of the currents could only explain 5.4% of the total variability in stomach contents (*p* < 0.01). Dragonfishes and lanternfishes were mainly preyed upon when currents had higher strength, while at medium/low currents the abundance of muscular squids in the stomachs increased (Figure 4C).

The SIMPER test showed that the highest average dissimilarity in BFT diet composition was determined by lanternfishes in significant comparisons between lunar phases, lunar irradiance and current strength levels (Tables S1–S3). Generally, lanternfishes and muscular squids together contributed to about 40% or more of dissimilarity between levels of all factors (Tables S1–S3), while dragonfishes contributed about 18–20% to the dissimilarity only in some comparisons between lunar phases (Table S1). 

The number of preys in the BFT stomachs varied significantly as the phases of the moon changed (χ^2^ = 34.746, df = 7, *p* = 1.249 × 10^−5^) (Figure S1). Pairwise comparison highlighted that “new moon”, “waning gibbous” and “full moon” were the lunar phases with highest number of preys in the BFT stomach, and these phases were significantly different with “waxing gibbous”, and only for the first two, even with “third quarter” (Figure S1). 

**Figure 5:**
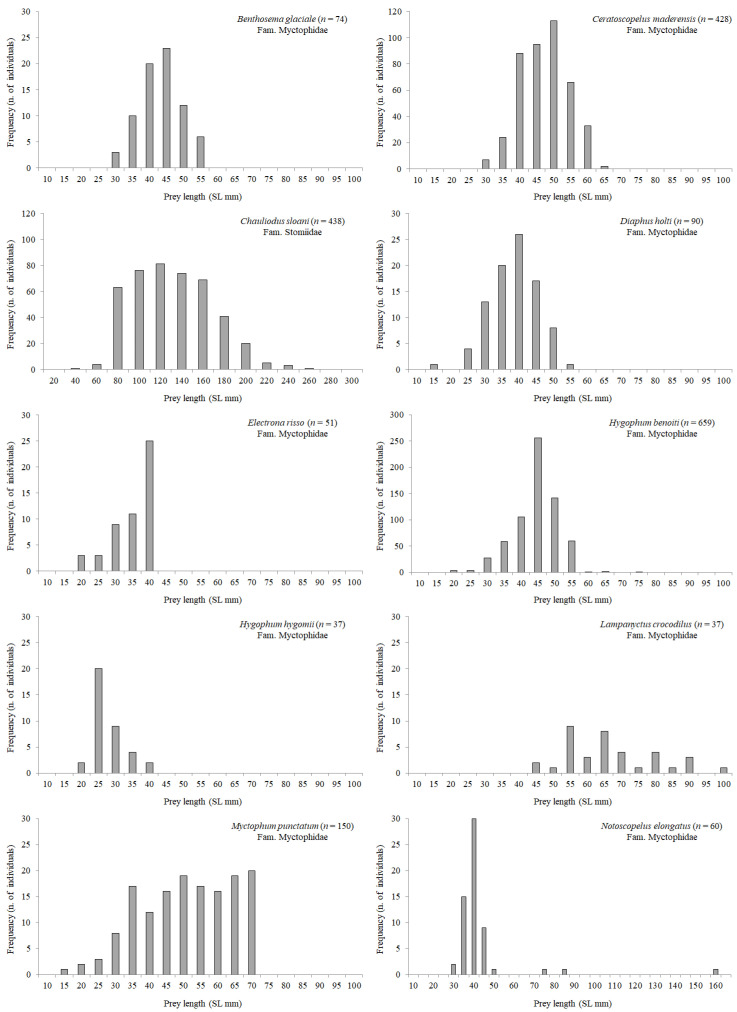
Length frequency distribution of the most abundant mesopelagic fish prey found in BFT stomachs in the Strait of Messina. Given the variability among species in size range and number of individuals, graphs do not report the same scale in x- and y-axes.

**Figure 6:**
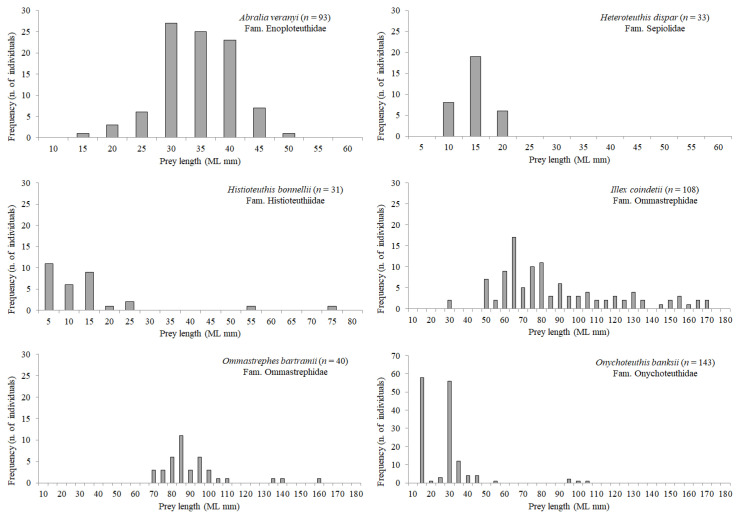
Length frequency distribution of the most abundant mesopelagic cephalopod prey found in BFT stomachs in the Strait of Messina. Given the variability among species in size range and number of individuals, graphs have different scaling on x- and y-axes.

Length frequency distribution of the most abundant mesopelagic fish and cephalopod prey species is shown in Figure 5 and Figure 6, respectively. In general, the size of most myctophid preys (*Benthosema glaciale*, *C. maderensis*, *Diaphus holti*, *Electrona risso*, *H. benoiti*, *Myctophum punctatum*) was ca. 30–55 mm SL, with few individuals belonging to the 55–70 mm SL classes, given that this length coincides for several Mediterranean lanternfish with their last stage of life. However, a few large individuals of *Lampanyctus crocodilus* and *Notoscopelus elongatus* (species which attain a larger size) were also found in the stomachs. The largest mesopelagic fish species preyed by BFT was *C. sloani*, ranging from 40.2 mm to 257.6 mm SL. Among cephalopods (Figure 6), *Illex coindetii* showed the highest variability in mantle length, ranging from 29.0 to 172.0 mm ML.

**Figure 7:**
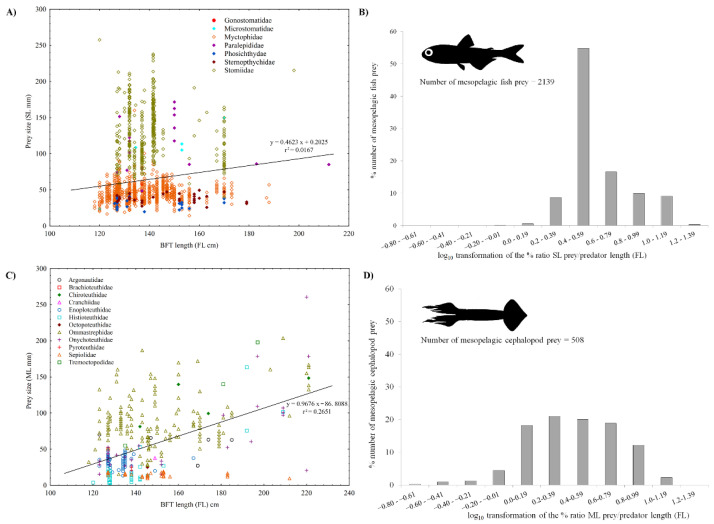
Relationship between the BFT fork length (FL cm) and the size of mesopelagic fish (**A**) and cephalopod (**C**) prey. Standard length (SL mm) and mantle length (ML mm) were considered for fish and cephalopod food items, respectively. Linear regression equations are also given. The histograms in (**B**,**D**) show the log10 transformation of the percent ratio between mesopelagic prey length and tuna fork length, for fish and cephalopods, respectively.

Figure 7 shows the relationship between BFT fork length and the size of the most important mesopelagic fish (Figure 7A) and cephalopod (Figure 7B) preys, grouped by families, in the area of the Strait of Messina. Estimated prey sizes were highly variable in relation to predator size, indicating highly opportunistic feeding: there was no significant association (r^2^ = 0.02) between mesopelagic fish prey size and BFT length (Figure 7A), and a low relationship between cephalopod prey and tuna sizes (r^2^ = 0.26) (Figure 7C). The histograms in Figure 7B,D show the log_10_ transformation of the percent ratio between mesopelagic prey length and BFT fork length, for fish and cephalopods, respectively. The log_10_ transformed data had a mean value of 0.6 for the mesopelagic fish prey, a standard deviation (SD) of 0.2 and a coefficient of variation (CV) of 0.4. On the contrary, the coefficient of variation for cephalopods was higher (CV = 0.8) and the average was smaller (mean = 0.4; SD = 0.3) than fish values, perhaps because the mantle length was used instead of total length. 

## 4. Discussion

Our findings indicate that the lunar cycle affects the diet composition of BFT in the study area as well as the trophic interaction between BFT and mesopelagic food resources. The data analyses showed that moon phases explained 15% of the total variance in prey composition found in BFT stomach contents, indicating that other environmental and ecological factors may also contribute to this variability in the study area. 

Our analyses also considered two moon-related factors, i.e., lunar irradiance and current strength. In our first hypothesis, we postulated that higher levels of moonlight irradiance would increase the success of visual predation by BFT on mesopelagic food resources (i.e., the predator has the chance to feed deeper in the water column), although higher light levels may reduce the extent of diel vertical migration of micronekton; on the other hand, during darker nights, this limit to the migration would be reduced, and micronekton would increase the extent of vertical excursions, allowing the BFT to forage more frequently on these prey items. Our results demonstrated that both cases occurred. Indeed, at the lowest levels of lunar irradiance, the abundance of mesopelagic fish and cephalopods in BFT stomachs increased when compared with samples obtained at moderate irradiance levels (e.g., Figure 2). Furthermore, we also observed high values of mesopelagic prey in BFT stomachs during the brightest moon phases (more than in days with moderate light intensity, but less than in the darkest days of lunar cycle), when BFT can explore a larger portion of the water column performing visual predation.

In the Strait of Messina, the indirect effect of the lunar cycle on the strength of currents and then on the upwelling amplitude also affects the BFT prey composition. As a result, the higher abundance of prey in BFT stomachs during new and full moon periods may also be due to an increased transport of food resources within the Strait of Messina, due to a more intense hydrodynamic regime, during specific phases of the lunar cycle. Indeed, moon phases regulate the intensity of tidal currents and have a direct effect on the increase in the upwelling phenomena in the study area: according to Mosetti [35], the intensity of tidal currents in the Strait of Messina is related to lunar cycle, being stronger during new moon and full moon phases, and weaker during the quarters, although it is also influenced by wind drift, meteorological surges and atmospheric pressure. Strong currents generated produce an increase in the passive transport of mesopelagic fauna to shallow waters at night [10,30]. 

**Figure 8:**
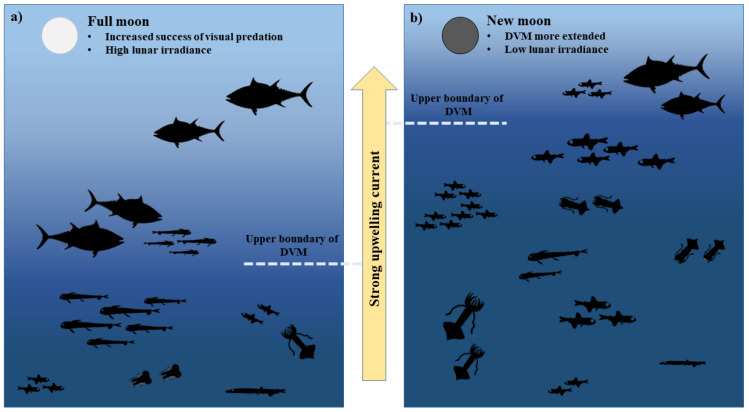
Schematic representation of BFT predation on mesopelagic resources in the Strait of Messina during full moon and new moon. In both lunar phases, the strong currents foster upwelling phenomena and, then, passive transport of mesopelagic food resources. On one hand, during full moon the high lunar irradiance increases the success of BFT visual predation (**a**). On the other hand, during the new moon the vertical movements of mesopelagic resources are larger and reach shallower water layers, making food more readily available for BFT (**b**).

These observations are summarized in Figure 8.

These results are also confirmed by two recent studies [10,30]. Battaglia et al. [30], analyzing the peculiar phenomenon of the stranding of mesopelagic fish in the Straits of Messina, found that the occurrence of these species also varied depending on moon phases, relating the stranding events to the extent of upwelling phenomena. In this study, the stranding of mesopelagic fish was highest during the new moon (coinciding with the lowest lunar irradiance and strongest currents) whereas the moon phase third quarter (lowest currents and moderate value of lunar irradiance) coincided with lower values of mesopelagic fish abundance. However, as observed for the stranding events in the Strait of Messina [30], the abundance of mesopelagic fish in the study area, and consequently the availability of these food resources to predators, may also depend on other factors not considered in this study. Indeed, according to Battaglia et al. [30], strong winds blowing from east and south-east strengthen the upwelling current and the transport of mesopelagic organisms in this area. Moreover, the lunar irradiance during night may be affected by the possible presence of clouds (even if this event does not influence the irradiance at new moon, i.e., the darkest moon phase). 

Evidence of the influence of lunar phases on the upwelling amplitude and then on the trophic interactions between predators and mesopelagic prey in the Strait of Messina is provided by Battaglia et al. [10], which investigated the feeding behavior of the blue jack mackerel *Trachurus picturatus* in this area. Battaglia et al. [10] found that *T. picturatus* consumed the highest rates of mesopelagic prey during the new moon and the full moon phases (i.e., when the strongest currents occur), and lowest rates during quarters (i.e., when current strength is reduced), suggesting that the lunar cycle plays a key role in regulating the transport of mesopelagic organisms toward the surface layers by modulating the intensity of currents. 

Our results showed a relationship between BFT foraging and the lunar cycle. We believe this result is due to interactions between the mesopelagic fish and squid communities and the effects of lunar phase-related variations in irradiance, local current strength and upwelling intensity. Further resolving the mechanisms associated with these results could be achieved by additional process-oriented studies. For example, direct sampling of the vertical distribution of the mesopelagic fish and squid community under different moon phase conditions using either fishing nets or acoustic methods could help to resolve the roles of illumination and current strength on the vertical distribution of potential prey species and their vulnerability to predators. Furthermore, similar sampling in an area less likely to be influenced by the lunar-induced tidal currents (i.e., outside the Strait of Messina) could also reveal how lunar phase variations in illumination affect the vertical distribution of the mesopelagic species without the influence of the lunar-related variations in sea currents. New tagging studies of BFT in the Strait of Messina and nearby areas could also help estimate the vertical behavior of these predators under different lunar phases and upwelling conditions. To date, there are no similar studies on BFT feeding activity and variability in relation to lunar cycle in other upwelling areas. The comparison of the potential distribution of mesopelagic species and BFT dietary composition in other upwelling areas could reveal potential co-occurring and/or distinct ecological processes. 

As noted above, there are likely other environmental and ecological factors that affect the prey composition of BFT diets in the study area. For instance, different species have different camouflage abilities and preferences for different isolumes [15,65], which could affect vulnerability of different species to visual foragers such as BFT. In general, detailed knowledge of the vertical migration behavior of mesopelagic fish and squids is limited, but one recent study indicates that it can be quite complex (e.g., discontinuous and intermittent through the night [11]). Furthermore, according to Battaglia et al. [30], mesopelagic fish with a lower swimming ability (e.g., some weakly migrant mesopelagic fish) are more vulnerable than others to the passive transport of upwelling currents in the Strait of Messina, consequently becoming more abundant in shallow waters when tidal currents become strong. Such species could be more likely to be encountered by predators such as BFT. 

On the basis of current ecological knowledge [66,67,68,69,70], the BFT diet in the study area included both highly vertically migrating mesopelagic fish (e.g., *M. punctatum*, *H. benoiti*), weakly migrant (e.g., *C. sloani*, *Diaphus holti*, *Electrona risso*) and not migrant species (e.g., adults of *Lampanyctus crocodilus*, *Symbolophorus veranyi*). These resources are primarily targeted by *T. thynnus* in the Strait of Messina because it is an opportunistic predator, which usually feeds on aggregated prey in schools or patches. Mesopelagic fish also attract several cephalopod predators, which follow them during the nychthemeral migration and became, in turn, prey of tunas. *O. banksii*, *I. coindetii* and *A. veranyi* were the most common cephalopods in the stomach content of *T. thynnus* in the study area. It is known that *O. banksii* can be also found at 4000 m depth, but appears in upper waters during night [71]. *I. coindetii* also performs vertical migrations, even though it prefers to remain below the thermocline [72], whereas *A. veranyi* lives in deeper waters and is also considered a diel vertical migrator [71]. The upwelling currents in the Strait of Messina play an important role in concentrating deep food resources in a restricted area, facilitating tuna foraging in shallow waters, although it is also known that BFT is able to carry out extensive depth excursions up to 1000 m in order to find its prey [19,20,73,74,75,76].

The analysis of the stomach contents of *T. thynnus*, caught in the Strait of Messina, indicated an important role of mesopelagic prey in the diet of this predator during its reproductive migration in the central Mediterranean. The eastern Atlantic and Mediterranean BFT stock shows fidelity to natal spawning grounds in the Mediterranean, returning during the reproductive period [73,77]. According to Battaglia et al. [25], the Strait of Messina is an important foraging area for *T. thynnus* during the prespawning and spawning periods, where, thanks to upwelling currents driven by geomorphological and hydrographic features as well as tidal currents (in particular conditions such as during new and full moons, current velocities that can reach 5 m/s; Mosetti [35]), a high concentration of mesopelagic resources can be observed [10,30,78]. For this reason, the Strait of Messina probably represents a key foraging area prior to spawning for *T. thynnus*, where this predator can restore energy spent during the migration by feeding on lipid-rich prey. Moreover, the predation on high energy-density prey relatively close to spawning may allow *T. thynnus* to fulfill its energetic requirements for ensuring successful gonadal development. According to Mourente et al. [79], lipid utilization in tuna metabolism is essential for the biosynthesis of gonadal constituents and useful to provide energy for swimming during the reproductive migration. In this study, the analysis of the stomach contents revealed that *T. thynnus* frequently consumed mesopelagic fish prey, especially lanternfish (such as *H. benoiti*, *C. maderensis* and *M. punctatum*) and the stomiid *C. sloani*, that in general are considered food resources with a high lipid content [80,81,82]. Although mesopelagic cephalopods were less abundant among prey items, they also contributed to the BFT energy intake, having been found with a high frequency of occurrence. In particular, muscle tissue of the most frequent squid prey (e.g., ommastrephid and onychoteuthid) is rich in proteins [83]. 

The food resource availability in the Strait of Messina and the mesopelagic forage accumulation due to the upwelling currents [10,30,67] may be the main reasons for the presence of adult tunas in this area throughout the year, supporting the hypothesis that a BFT subpopulation might not migrate toward the Atlantic Ocean [43,44,45] and exploit this area as an overwintering foraging ground. The fronts generated by upwelling currents of the Strait of Messina represent hotspots of primary and secondary production [41], where BFT may find optimal trophic conditions for foraging on mesopelagic prey. Other studies, based on satellite tagging, have recently observed the importance of the Mediterranean Sea not only for spawning, but also as a significant foraging area during overwintering periods [84,85,86,87].

Mesopelagic food resources are targeted by adult BFT also in other areas. A recent study reported a large amount of mesopelagic prey in BFT stomach contents in Icelandic waters, especially cephalopods (*Todarodes sagittatus* and *Gonatus* sp.) and fish (barracudinas, Paralepididae), inferring an extensive diving and feeding behavior in the mesopelagic layer [27]. The stomiid *C. sloani* was the most important prey in the upwelling area of the Strait of Gibraltar, but other mesopelagic fish (e.g., Myctophidae) and cephalopods (e.g., Ommastrephidae, Histioteuthidae) were also found in BFT stomach contents in this area [88]. The dominance of mesopelagic food resources was also observed by Karakulak et al. [29] in the eastern Mediterranean Sea, where tunas preyed mostly on lanternfish (Myctophidae) and pelagic octopuses (Argonautida). Interestingly, *H. benoiti* was the most abundant lanternfish prey in both the eastern Mediterranean Sea [29] and the Strait of Messina ([25]; present study).

Our findings add further to the literature on effects of lunar variations on marine life. We have seen significant differences in diet composition of a top predator among different lunar phases and that these effects are due to lunar-related variations in both illumination and local currents and upwelling intensity. These effects alter vulnerabilities of especially the mesopelagic fish and squid communities to predation while simultaneously enabling the predator to encounter an energy-rich food source at shallow depth.

## 5. Conclusions

In conclusion, this study sheds light for the first time on the influence of the lunar cycle on the foraging activity of BFT in a Mediterranean upwelling area (Strait of Messina), characterized by the presence of abundant mesopelagic fauna that represents a fundamental and strategic food source for this predator. The analysis of the trophic interaction of BFT with mesopelagic fishes and cephalopods allowed us to focus on how the lunar cycle may influence the availability of mesopelagic prey for this predator, not only in relation to different moon phases, but also considering two potential impacts of the lunar cycle: lunar irradiance and current strength. 

The Strait of Messina represents an important feeding ground for BFT, especially during the pre-reproductive period, due to its unique hydrodynamic and biological characteristics, which support the trophic web and make a high biomass of mesopelagic resources available to predators. The abundance and concentration of these preys are highly dependent on upwelling currents, which in turn are influenced by the lunar cycle (new and full moon with strong currents, quarters with weaker currents). Furthermore, lunar irradiance contributes to the variation of prey abundance and composition, increasing the success of visual predation on mesopelagic resources at high light levels (i.e., full moon) in the water column. The higher abundance of mesopelagic food items in BFT stomachs was observed near new and full moon, probably due to an intense transport of prey from deep waters, caused by a more intense hydrodynamic regime in these lunar phases. Although it was demonstrated that moon phases explain 15% of the total variance in prey composition, other environmental and ecological factors may influence the prey abundance and distribution in the water column of the Strait of Messina. It would be interesting to relate vertical migrations of these prey resources to vertical and horizontal movements of BFT, integrating echo-survey to satellite tagging experiments. 

The study period (March–June) was not precisely the peak feeding season for adult bluefin tuna, since this peak usually occurs after spawning (July–November). According to Cort and Estruch [89], during this period the condition factor (K) increases progressively. Therefore, it would be useful in the future to extend this dietary study to the peak feeding season of bluefin tuna in the Strait of Messina and adjacent areas, comparing the results in relation to the lunar phases.

Recently, some studies underlined the importance of mesopelagic prey resources for BFT in different foraging areas [25,27,29], probably also due to the recent decrease in small epipelagic fish such as sardines and anchovies [90,91,92,93], which were an important component in BFT diet. Therefore, for the purpose of conservation and management of BFT resources, it is important to implement more investigations to better understand the opportunistic behavior of this species. The recent attempt to exploit mesopelagic resources by pelagic fisheries (e.g., [94,95,96,97]) should take into account that these organisms are key prey for BFT but also other large predators (marine mammals, swordfish, sharks); ecosystem-based approaches to fishery management that accommodate predator–prey dynamics and predator foraging needs may need to be developed further. 

## Figures and Tables

**Table 1:** Percentage abundance (%N) and frequency of occurrence (%F) of BFT mesopelagic cephalopod and fish prey (total values for other invertebrates and fish are also given). The column “moon phases” indicates the period of lunar cycle in which each prey was found (a: new moon; b: waxing crescent; c: first quarter; d: waxing gibbous; e: full moon; f: waning gibbous; g: third quarter; h: waning crescent).

Food Category/Family	Species	N%	F%	Moon Phases
**Mesopelagic cephalopods**		**11.79**	**63.79**	**a, b, c, d, e, f, g, h**
Argonautidae	*Argonauta argo*	0.18	5.17	a, c, f
Brachioteuthidae	*Brachioteuthis riisei*	0.02	0.86	a
Chiroteuthidae	*Chiroteuthis veranyi*	0.09	3.45	f, g
Cranchiidae	*Galiteuthis armata*	0.02	0.86	c
Enoploteuthidae	*Abralia veranyi*	2.11	6.90	a, c, e, f, h
	*Abraliopsis morisii*	0.16	4.31	b, c, e, f, h
Histioteuthidae	*Histioteuthis bonnellii*	0.70	8.62	a, c, e, f, g
	*Histioteuthis reversa*	0.14	3.45	a, g
Octopoteuthidae	*Octopoteuthis sicula*	0.02	0.86	a
Ommastrephidae	*Illex coindetii*	2.59	36.21	a, b, c, d, e, f, g, h
	*Ommastrephes bartramii*	0.95	8.62	c, d, e, f, g, h
	*Todarodes sagittatus*	0.48	9.48	a, b, c, e, f, g
Onycoteuthidae	*Ancistroteuthis lichtensteinii*	0.16	5.17	a, d, g
	*Onychoteuthis banksii*	3.25	11.21	a, b, c, d, e, f, g, h
Pyroteuthidae	*Pyroteuthis margaritifera*	0.05	0.86	f
Sepiolidae	*Heteroteuthis dispar*	0.75	11.21	a, b, c, d, f, g, h
Thysanoteuthidae	*Thysanoteuthis rhombus*	0.02	0.86	g
Tremoctopodidae	*Tremoctopus violaceus*	0.07	2.59	g, h
	Teuthida ind	0.02	0.86	f
**Mesopelagic fish**		**48.92**	**65.52**	**a, b, c, d, e, f, g, h**
Gonostomatidae	*Gonostoma denudatum*	0.05	1.72	d, e
Microstomatidae	*Microstoma microstoma*	0.09	2.59	a, d, f
	*Nansenia oblita*	0.05	1.72	b, c
Myctophidae	*Benthosema glaciale*	1.68	9.48	a, b, c, e, f
	*Ceratoscopelus maderensis*	9.75	37.93	a, b, c, d, e, f, h
	*Diaphus holti*	2.04	13.79	a, b, d, e, f
	*Diaphus rafinesquii*	0.07	0.86	a
	*Electrona risso*	1.16	6.90	a, b, c, d, f
	*Hygophum benoiti*	15.02	37.93	a, b, c, d, e, f, h
	*Hygophum hygomii*	0.84	8.62	a, b, c
	*Lampanyctus crocodilus*	0.82	6.90	a, e, f
	*Lampanyctus pusillus*	0.32	5.17	a, b, f
	*Myctophum punctatum*	3.43	26.72	a, b, c, e, f, h
	*Notoscopelus elongatus*	1.36	10.34	a, b, c, e, f
	*Symbolophorus veranyi*	0.02	0.86	e
Paralepididae	*Arctozenus risso*	0.14	4.31	b, c
	*Paralepis coregonoides*	0.23	4.31	a, b, d, f, g
	*Paralepis speciosa*	0.02	0.86	g
	*Sudis hyalina*	0.07	2.59	b, e, f
Phosichthyidae	*Ichthyococcus ovatus*	0.09	0.86	f
	*Vinciguerria attenuata*	0.61	6.90	a, b, c, e
	*Vinciguerria poweriae*	0.07	1.72	a, f
Sternoptychidae	*Argyropelecus hemygimnus*	0.09	2.59	a, b, f
	*Maurolicus muelleri*	0.61	13.79	a, b, c, e, f
Stomiidae	*Chauliodus sloani*	10.13	21.55	a, b, d, e, f
	*Stomias boa boa*	0.16	4.31	a, e, f
**Other invertebrates**		**35.97**	**51.72**	**a, b, c, d, e, f, g, h**
**Other fish**		**3.32**	**46.55**	**a, b, c, d, e, f, g, h**

## Data Availability

Not applicable.

## Supplementary Materials

The following supporting information can be downloaded at: https://www.mdpi.com/article/10.3390/ani12172261/s1, Figure S1: Variations in the number of preys in BFT stomach contents, between different moon phases, Table S1: SIMPER of prey groups contributing (%) to dissimilarity among levels of the factor “Moon phase”, Table S2: SIMPER of prey groups contributing (%) to dissimilarity among levels of the factor “Lunar irradiance”, Table S3: SIMPER of prey groups contributing (%) to dissimilarity among levels of the factor “Current strength”.
