# Supplementary material for: Importance of the Lunar Cycle on Mesopelagic Foraging by Atlantic Bluefin Tuna in the Upwelling Area of the Strait of Messina (Central Mediterranean Sea)
Source: Animals (Basel). 2022 Aug 31;12(17):2261. doi: 10.3390/ani12172261 (PMC9454512; doi:10.3390/ani12172261)
Supplement: Supplementary file 1 [file animals-12-02261-s001.zip › animals-1873289-supplementary.pdf]

SUPPLEMENTARY MATERIALS

Battaglia et al. - Importance of lunar cycle on mesopelagic foraging by Atlantic bluefin tuna in the upwelling area of the Strait of Messina (central Mediterranean Sea)

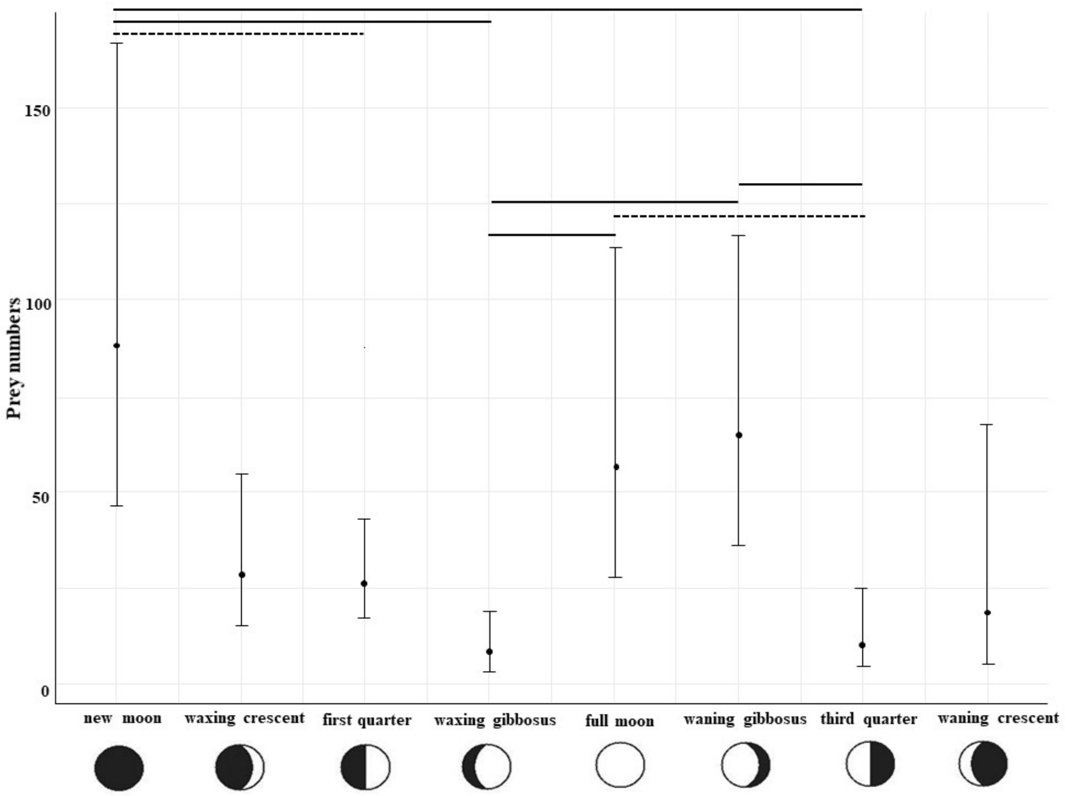

Figure S1. Variations in the number of prey in BFT stomach contents, between different moon phases. Continuous lines indicate significant difference with $p < 0.05$; dashed lines indicate a $p < 0.1$.

Table S1. SIMPER of prey groups contributing (%) to dissimilarity among levels of the factor "Moon phase".

**third quarter vs waning gibbosus**	**overall = 0.87**						
**species**	**average**	**sd**	**ratio**	**ava**	**avb**	**Cumsum (%)**	**Contribution (%)**
Other invertebrates	0.25	0.30	0.84	6.91	27.33	28.48	28.48
Muscular squids	0.16	0.22	0.74	1.73	2.67	47.36	18.88
Other fish	0.16	0.23	0.69	1.73	1.38	65.45	18.08
Lanternfishes	0.14	0.26	0.56	0.00	15.14	81.98	16.53
Dragonfishes	0.06	0.09	0.63	0.00	4.71	88.35	6.38
Bouyant squids	0.03	0.06	0.62	0.27	1.14	92.29	3.93
Other mesopelagic fish	0.03	0.09	0.37	0.18	0.81	95.91	3.62
Pelagic octopods	0.02	0.07	0.33	0.18	0.05	98.64	2.73
Sepiolids	0.01	0.03	0.42	0.45	0.29	100.00	1.36

**third quarter vs waning crescent**	**overall = 0.81**						
**species**	**average**	**sd**	**ratio**	**ava**	**avb**	**Cumsum (%)**	**Contribution (%)**
Muscular squids	0.39	0.34	1.15	1.73	7.00	48.37	48.37
Other invertebrates	0.21	0.27	0.79	6.91	0.86	74.77	26.40
Other fish	0.11	0.21	0.53	1.73	0.57	88.86	14.08
Pelagic octopods	0.03	0.10	0.32	0.18	0.14	92.80	3.95
Sepiolids	0.02	0.03	0.54	0.45	0.43	95.12	2.32
Lanternfishes	0.02	0.03	0.57	0.00	0.57	97.36	2.23
Bouyant squids	0.02	0.04	0.45	0.27	0.00	99.55	2.19
Other mesopelagic fish	0.00	0.01	0.45	0.18	0.00	100.00	0.45
Dragonfishes	0.00	0.00	NA	0.00	0.00	100.00	0.00

**third quarter vs new moon**	**overall = 0.87**						
**species**	**average**	**sd**	**ratio**	**ava**	**avb**	**Cumsum (%)**	**Contribution (%)**
Other invertebrates	0.31	0.26	1.22	6.91	37.19	36.07	36.07
Lanternfishes	0.19	0.19	1.00	0.00	26.25	57.60	21.53
Other fish	0.13	0.20	0.63	1.73	1.56	72.45	14.85
Muscular squids	0.13	0.17	0.76	1.73	4.06	87.17	14.72
Dragonfishes	0.04	0.10	0.36	0.00	3.19	91.36	4.19
Sepiolids	0.03	0.07	0.38	0.45	0.75	94.46	3.11
Pelagic octopods	0.02	0.05	0.36	0.18	0.13	96.46	2.00
Other mesopelagic fish	0.02	0.03	0.52	0.18	1.63	98.38	1.92
Bouyant squids	0.01	0.03	0.52	0.27	0.38	100.00	1.62

**third quarter vs waxing crescent**	**overall = 0.85**						
**species**	**average**	**sd**	**ratio**	**ava**	**avb**	**Cumsum (%)**	**Contribution (%)**
Other invertebrates	0.28	0.27	1.04	6.91	10.69	32.52	32.52
Lanternfishes	0.26	0.28	0.92	0.00	9.13	63.09	30.56
Muscular squids	0.15	0.19	0.80	1.73	2.94	80.97	17.88
Other fish	0.07	0.12	0.59	1.73	0.69	89.11	8.14
Other mesopelagic fish	0.04	0.05	0.81	0.18	1.00	94.11	5.00
Sepiolids	0.02	0.03	0.51	0.45	0.25	95.92	1.81
Pelagic octopods	0.01	0.03	0.40	0.18	0.06	97.54	1.63
Dragonfishes	0.01	0.03	0.40	0.00	0.56	98.84	1.30
Bouyant squids	0.01	0.02	0.49	0.27	0.00	100.00	1.16

**third quarter vs first quarter**	**overall = 0.88**						
**species**	**average**	**sd**	**ratio**	**ava**	**avb**	**Cumsum (%)**	**Contribution (%)**
Lanternfishes	0.21	0.33	0.64	0.00	12.38	24.45	24.45
Muscular squids	0.21	0.27	0.78	1.73	4.59	48.68	24.23
Other invertebrates	0.21	0.27	0.79	6.91	1.12	72.90	24.21
Other fish	0.16	0.25	0.63	1.73	0.94	90.64	17.74
Pelagic octopods	0.03	0.07	0.36	0.18	0.12	93.69	3.05
Other mesopelagic fish	0.03	0.10	0.26	0.18	0.21	96.71	3.03
Bouyant squids	0.02	0.04	0.43	0.27	0.06	98.86	2.14
Sepiolids	0.01	0.03	0.39	0.45	0.06	100.00	1.14
Dragonfishes	0.00	0.00	NA	0.00	0.00	100.00	0.00

**third quarter vs waxing gibbosus**	**overall = 0.91**						
**species**	**average**	**sd**	**ratio**	**ava**	**avb**	**Cumsum (%)**	**Contribution (%)**
Other invertebrates	0.26	0.29	0.90	6.91	0.75	28.83	28.83
Muscular squids	0.25	0.24	1.02	1.73	3.00	55.91	27.08
Other fish	0.19	0.28	0.69	1.73	0.63	77.23	21.32
Lanternfishes	0.08	0.15	0.51	0.00	1.13	85.60	8.37
Other mesopelagic fish	0.07	0.14	0.47	0.18	1.00	92.98	7.38
Pelagic octopods	0.02	0.07	0.35	0.18	0.00	95.69	2.71
Bouyant squids	0.02	0.04	0.49	0.27	0.00	97.63	1.94
Sepiolids	0.01	0.03	0.48	0.45	0.13	99.11	1.47
Dragonfishes	0.01	0.02	0.34	0.00	0.13	100.00	0.89

**third quarter vs full moon**	**overall = 0.87**						
**species**	**average**	**sd**	**ratio**	**ava**	**avb**	**Cumsum (%)**	**Contribution (%)**
Other invertebrates	0.21	0.22	0.93	6.91	7.80	23.93	23.93
Lanternfishes	0.20	0.23	0.88	0.00	12.67	46.91	22.97
Dragonfishes	0.17	0.27	0.62	0.00	19.53	66.19	19.28
Muscular squids	0.12	0.22	0.53	1.73	1.07	79.62	13.43
Other fish	0.11	0.18	0.59	1.73	1.40	91.88	12.26
Other mesopelagic fish	0.03	0.09	0.38	0.18	1.13	95.78	3.90
Pelagic octopods	0.02	0.06	0.24	0.18	0.00	97.52	1.73
Bouyant squids	0.01	0.03	0.48	0.27	0.53	99.16	1.64
Sepiolids	0.01	0.02	0.34	0.45	0.00	100.00	0.84

**waning gibbosus vs waning crescent**	**overall = 0.86**						
**species**	**average**	**sd**	**ratio**	**ava**	**avb**	**Cumsum (%)**	**Contribution (%)**
Muscular squids	0.27	0.28	0.95	2.67	7.00	31.21	31.21
Lanternfishes	0.16	0.27	0.60	15.14	0.57	49.89	18.69
Other invertebrates	0.15	0.26	0.59	27.33	0.86	67.69	17.80
Other fish	0.14	0.22	0.61	1.38	0.57	83.57	15.88
Dragonfishes	0.06	0.09	0.61	4.71	0.00	90.30	6.73
Other mesopelagic fish	0.03	0.09	0.34	0.81	0.00	93.97	3.67
Bouyant squids	0.03	0.06	0.47	1.14	0.00	97.24	3.27
Sepiolids	0.01	0.03	0.45	0.29	0.43	98.68	1.45
Pelagic octopods	0.01	0.05	0.23	0.05	0.14	100.00	1.32

waning gibbosus vs new moon **overall = 0.85**

species	average	sd	ratio	ava	avb	Cumsum (%)	Contribution (%)
Other invertebrates	0.27	0.26	1.04	27.33	37.19	31.77	31.77
Lanternfishes	0.24	0.22	1.10	15.14	26.25	59.75	27.98
Muscular squids	0.10	0.15	0.70	2.67	4.06	72.07	12.32
Other fish	0.09	0.16	0.58	1.38	1.56	83.27	11.21
Dragonfishes	0.06	0.10	0.62	4.71	3.19	90.77	7.49
Other mesopelagic fish	0.03	0.06	0.46	0.81	1.63	94.15	3.38
Sepiolids	0.02	0.06	0.33	0.29	0.75	96.62	2.47
Bouyant squids	0.02	0.04	0.56	1.14	0.38	98.93	2.31
Pelagic octopods	0.01	0.03	0.27	0.05	0.13	100.00	1.07

**waning gibbosus vs waxing crescent**	**overall = 0.85**						
**species**	**average**	**sd**	**ratio**	**ava**	**avb**	**Cumsum (%)**	**Contribution (%)**
Lanternfishes	0.30	0.27	1.10	15.14	9.13	35.82	35.82
Other invertebrates	0.22	0.28	0.77	27.33	10.69	61.50	25.68
Muscular squids	0.14	0.18	0.74	2.67	2.94	77.64	16.14
Other fish	0.06	0.09	0.68	1.38	0.69	85.06	7.43
Dragonfishes	0.05	0.06	0.76	4.71	0.56	90.53	5.47
Other mesopelagic fish	0.05	0.06	0.75	0.81	1.00	95.91	5.38
Bouyant squids	0.02	0.03	0.57	1.14	0.00	97.91	2.01
Sepiolids	0.01	0.03	0.41	0.29	0.25	99.15	1.24
Pelagic octopods	0.01	0.03	0.27	0.05	0.06	100.00	0.85

**waning gibbosus vs first quarter**	**overall = 0.86**						
**species**	**average**	**sd**	**ratio**	**ava**	**avb**	**Cumsum (%)**	**Contribution (%)**
Lanternfishes	0.29	0.33	0.87	15.14	12.38	33.97	33.97
Muscular squids	0.15	0.23	0.67	2.67	4.59	51.78	17.81
Other invertebrates	0.14	0.25	0.58	27.33	1.12	68.59	16.81
Other fish	0.13	0.21	0.64	1.38	0.94	84.02	15.42
Dragonfishes	0.05	0.08	0.62	4.71	0.00	90.08	6.06
Other mesopelagic fish	0.04	0.11	0.39	0.81	0.21	94.95	4.87
Bouyant squids	0.03	0.05	0.51	1.14	0.06	98.06	3.11
Pelagic octopods	0.01	0.05	0.25	0.05	0.12	99.45	1.39
Sepiolids	0.00	0.02	0.28	0.29	0.06	100.00	0.55

**waning gibbosus vs waxing gibbosus**	**overall = 0.85**						
**species**	**average**	**sd**	**ratio**	**ava**	**avb**	**Cumsum (%)**	**Contribution (%)**
Lanternfishes	0.20	0.26	0.77	15.14	1.13	23.40	23.40
Muscular squids	0.17	0.21	0.78	2.67	3.00	43.17	19.77
Other invertebrates	0.16	0.25	0.63	27.33	0.75	61.87	18.70
Other fish	0.14	0.22	0.64	1.38	0.63	78.54	16.67
Other mesopelagic fish	0.08	0.14	0.54	0.81	1.00	87.66	9.12
Dragonfishes	0.06	0.09	0.70	4.71	0.13	94.99	7.33
Bouyant squids	0.03	0.05	0.53	1.14	0.00	98.24	3.25
Pelagic octopods	0.01	0.04	0.20	0.05	0.00	99.14	0.90
Sepiolids	0.01	0.02	0.39	0.29	0.13	100.00	0.86

**waning gibbosus vs full moon**	**overall = 0.85**						
**species**	**average**	**sd**	**ratio**	**ava**	**avb**	**Cumsum (%)**	**Contribution (%)**

Lanternfishes	0.26	0.25	1.02	15.14	12.67	30.25	30.25
Dragonfishes	0.18	0.25	0.71	4.71	19.53	51.14	20.89
Other invertebrates	0.18	0.23	0.76	27.33	7.80	71.88	20.74
Other fish	0.09	0.17	0.54	1.38	1.40	82.54	10.66
Muscular squids	0.08	0.15	0.52	2.67	1.07	91.61	9.07
Other mesopelagic fish	0.04	0.10	0.43	0.81	1.13	96.61	5.00
Bouyant squids	0.02	0.04	0.50	1.14	0.53	99.09	2.48
Pelagic octopods	0.00	0.03	0.14	0.05	0.00	99.62	0.53
Sepiolids	0.00	0.01	0.23	0.29	0.00	100.00	0.38

**waxing gibbosus vs full moon**	**overall = 0.88**						
**species**	**average**	**sd**	**ratio**	**ava**	**avb**	**Cumsum (%)**	**Contribution (%)**
Lanternfishes	0.23	0.23	1.01	1.13	12.67	26.21	26.21
Dragonfishes	0.18	0.28	0.64	0.13	19.53	46.33	20.12
Other invertebrates	0.16	0.20	0.81	0.75	7.80	65.08	18.75
Muscular squids	0.12	0.19	0.62	3.00	1.07	78.44	13.36
Other fish	0.12	0.21	0.54	0.63	1.40	91.58	13.14
Other mesopelagic fish	0.07	0.13	0.50	1.00	1.13	99.25	7.67
Bouyant squids	0.00	0.01	0.27	0.00	0.53	99.68	0.43
Sepiolids	0.00	0.01	0.33	0.13	0.00	100.00	0.32
Pelagic octopods	0.00	0.00	NA	0.00	0.00	100.00	0.00

**waxing gibbosus vs waning crescent**	**overall = 0.88**						
**species**	**average**	**sd**	**ratio**	**ava**	**avb**	**Cumsum (%)**	**Contribution (%)**
Muscular squids	0.35	0.31	1.14	3.00	7.00	40.03	40.03
Other fish	0.19	0.32	0.58	0.63	0.57	61.56	21.53
Other invertebrates	0.15	0.27	0.57	0.75	0.86	78.78	17.21
Lanternfishes	0.09	0.16	0.59	1.13	0.57	89.22	10.44
Other mesopelagic fish	0.07	0.15	0.44	1.00	0.00	96.99	7.77
Sepiolids	0.01	0.03	0.52	0.13	0.43	98.62	1.62
Dragonfishes	0.01	0.03	0.34	0.13	0.00	99.59	0.97
Pelagic octopods	0.00	0.01	0.40	0.00	0.14	100.00	0.41
Bouyant squids	0.00	0.00	NA	0.00	0.00	100.00	0.00

**waxing gibbosus vs new moon**	**overall = 0.85**						
**species**	**average**	**sd**	**ratio**	**ava**	**avb**	**Cumsum (%)**	**Contribution (%)**
Other invertebrates	0.26	0.24	1.09	0.75	37.19	31.01	31.01
Lanternfishes	0.20	0.18	1.11	1.13	26.25	55.25	24.24
Muscular squids	0.15	0.19	0.82	3.00	4.06	73.34	18.08
Other fish	0.09	0.15	0.63	0.63	1.56	84.50	11.16
Other mesopelagic fish	0.05	0.10	0.50	1.00	1.63	90.42	5.92
Dragonfishes	0.04	0.10	0.40	0.13	3.19	95.29	4.87
Sepiolids	0.02	0.07	0.35	0.13	0.75	98.23	2.94
Pelagic octopods	0.01	0.03	0.25	0.00	0.13	99.12	0.89
Bouyant squids	0.01	0.02	0.43	0.00	0.38	100.00	0.88

**waxing gibbosus vs waxing crescent**	**overall = 0.83**						
**species**	**average**	**sd**	**ratio**	**ava**	**avb**	**Cumsum (%)**	**Contribution (%)**
Lanternfishes	0.28	0.28	1.00	1.13	9.13	33.30	33.30

Muscular squids	0.20	0.22	0.92	3.00	2.94	57.62	24.32
Other invertebrates	0.20	0.26	0.76	0.75	10.69	81.23	23.61
Other mesopelagic fish	0.07	0.08	0.82	1.00	1.00	89.54	8.31
Other fish	0.05	0.07	0.73	0.63	0.69	95.98	6.43
Dragonfishes	0.02	0.03	0.52	0.13	0.56	97.87	1.89
Sepiolids	0.01	0.03	0.44	0.13	0.25	99.24	1.37
Pelagic octopods	0.01	0.03	0.24	0.00	0.06	100.00	0.76
Bouyant squids	0.00	0.00	NA	0.00	0.00	100.00	0.00

**waxing gibbosus vs first quarter**	**overall = 0.85**						
**species**	**average**	**sd**	**ratio**	**ava**	**avb**	**Cumsum (%)**	**Contribution (%)**
Lanternfishes	0.27	0.33	0.81	1.13	12.38	31.34	31.34
Muscular squids	0.22	0.26	0.82	3.00	4.59	56.79	25.45
Other fish	0.14	0.22	0.66	0.63	0.94	73.60	16.81
Other invertebrates	0.12	0.20	0.61	0.75	1.12	87.98	14.38
Other mesopelagic fish	0.08	0.15	0.53	1.00	0.21	97.05	9.08
Pelagic octopods	0.01	0.04	0.20	0.00	0.12	97.96	0.90
Dragonfishes	0.01	0.02	0.33	0.13	0.00	98.83	0.88
Bouyant squids	0.01	0.03	0.17	0.00	0.06	99.45	0.62
Sepiolids	0.00	0.01	0.40	0.13	0.06	100.00	0.55

**waning crescent vs new moon**	**overall = 0.89**						
**species**	**average**	**sd**	**ratio**	**ava**	**avb**	**Cumsum (%)**	**Contribution (%)**
Other invertebrates	0.26	0.25	1.03	0.86	37.19	28.74	28.74
Muscular squids	0.23	0.25	0.92	7.00	4.06	54.17	25.43
Lanternfishes	0.19	0.19	1.02	0.57	26.25	75.84	21.67
Other fish	0.12	0.22	0.55	0.57	1.56	89.14	13.31
Dragonfishes	0.04	0.10	0.36	0.00	3.19	93.27	4.13
Sepiolids	0.03	0.07	0.38	0.43	0.75	96.40	3.13
Other mesopelagic fish	0.02	0.03	0.47	0.00	1.63	98.14	1.73
Pelagic octopods	0.01	0.03	0.30	0.14	0.13	99.19	1.05
Bouyant squids	0.01	0.02	0.42	0.00	0.38	100.00	0.81

**waning crescent vs waxing crescent**	**overall = 0.86**						
**species**	**average**	**sd**	**ratio**	**ava**	**avb**	**Cumsum (%)**	**Contribution (%)**
Lanternfishes	0.27	0.29	0.92	0.57	9.13	30.89	30.89
Muscular squids	0.27	0.25	1.08	7.00	2.94	61.75	30.86
Other invertebrates	0.20	0.26	0.77	0.86	10.69	85.36	23.62
Other fish	0.05	0.07	0.70	0.57	0.69	90.71	5.34
Other mesopelagic fish	0.04	0.06	0.80	0.00	1.00	95.84	5.13
Sepiolids	0.02	0.03	0.52	0.43	0.25	97.68	1.84
Dragonfishes	0.01	0.03	0.40	0.00	0.56	98.98	1.31
Pelagic octopods	0.01	0.03	0.30	0.14	0.06	100.00	1.02
Bouyant squids	0.00	0.00	NA	0.00	0.00	100.00	0.00

**waning crescent vs first quarter**	**overall = 0.86**						
**species**	**average**	**sd**	**ratio**	**ava**	**avb**	**Cumsum (%)**	**Contribution (%)**
Muscular squids	0.33	0.31	1.04	7.00	4.59	37.95	37.95
Lanternfishes	0.23	0.34	0.68	0.57	12.38	64.85	26.90

Other fish	0.14	0.25	0.57	0.57	0.94	81.28	16.43
Other invertebrates	0.10	0.19	0.53	0.86	1.12	93.30	12.02
Other mesopelagic fish	0.03	0.13	0.23	0.00	0.21	96.87	3.56
Sepiolids	0.01	0.03	0.42	0.43	0.06	98.09	1.23
Pelagic octopods	0.01	0.04	0.26	0.14	0.12	99.31	1.22
Bouyant squids	0.01	0.04	0.15	0.00	0.06	100.00	0.69
Dragonfishes	0.00	0.00	NA	0.00	0.00	100.00	0.00

**waning crescent vs full moon**	**overall = 0.86**						
**species**	**average**	**sd**	**ratio**	**ava**	**avb**	**Cumsum (%)**	**Contribution (%)**
Muscular squids	0.21	0.28	0.74	7.00	1.07	24.46	24.46
Lanternfishes	0.21	0.23	0.91	0.57	12.67	48.61	24.15
Dragonfishes	0.17	0.28	0.62	0.00	19.53	68.38	19.77
Other invertebrates	0.14	0.17	0.83	0.86	7.80	84.36	15.98
Other fish	0.09	0.15	0.56	0.57	1.40	94.37	10.01
Other mesopelagic fish	0.03	0.09	0.37	0.00	1.13	98.38	4.01
Sepiolids	0.01	0.02	0.36	0.43	0.00	99.27	0.89
Bouyant squids	0.00	0.01	0.27	0.00	0.53	99.70	0.43
Pelagic octopods	0.00	0.01	0.36	0.14	0.00	100.00	0.30

**new moon vs waxing crescent**	**overall = 0.80**						
**species**	**average**	**sd**	**ratio**	**ava**	**avb**	**Cumsum (%)**	**Contribution (%)**
Other invertebrates	0.28	0.25	1.11	37.19	10.69	34.73	34.73
Lanternfishes	0.25	0.21	1.19	26.25	9.13	66.33	31.60
Muscular squids	0.11	0.14	0.75	4.06	2.94	79.84	13.51
Other fish	0.06	0.10	0.59	1.56	0.69	87.19	7.35
Dragonfishes	0.04	0.09	0.43	3.19	0.56	91.88	4.68
Other mesopelagic fish	0.03	0.04	0.78	1.63	1.00	95.97	4.10
Sepiolids	0.02	0.05	0.39	0.75	0.25	98.43	2.45
Pelagic octopods	0.01	0.02	0.30	0.13	0.06	99.36	0.93
Bouyant squids	0.01	0.01	0.44	0.38	0.00	100.00	0.64

**new moon vs first quarter**	**overall = 0.86**						
**species**	**average**	**sd**	**ratio**	**ava**	**avb**	**Cumsum (%)**	**Contribution (%)**
Lanternfishes	0.27	0.26	1.06	26.25	12.38	31.89	31.89
Other invertebrates	0.25	0.24	1.03	37.19	1.12	60.89	29.01
Muscular squids	0.14	0.20	0.68	4.06	4.59	76.87	15.98
Other fish	0.10	0.17	0.60	1.56	0.94	88.67	11.80
Dragonfishes	0.03	0.10	0.36	3.19	0.00	92.72	4.05
Other mesopelagic fish	0.02	0.05	0.44	1.63	0.21	95.39	2.67
Sepiolids	0.02	0.07	0.31	0.75	0.06	97.82	2.43
Pelagic octopods	0.01	0.04	0.28	0.13	0.12	99.00	1.18
Bouyant squids	0.01	0.02	0.37	0.38	0.06	100.00	1.00

**new moon vs full moon**	**overall = 0.83**						
**species**	**average**	**sd**	**ratio**	**ava**	**avb**	**Cumsum (%)**	**Contribution (%)**
Other invertebrates	0.24	0.22	1.11	37.19	7.80	28.73	28.73
Lanternfishes	0.23	0.19	1.21	26.25	12.67	56.57	27.85
Dragonfishes	0.15	0.24	0.64	3.19	19.53	74.79	18.22

Other fish	0.08	0.15	0.52	1.56	1.40	84.20	9.40
Muscular squids	0.07	0.13	0.57	4.06	1.07	93.12	8.93
Other mesopelagic fish	0.03	0.07	0.45	1.63	1.13	96.66	3.54
Sepiolids	0.02	0.05	0.28	0.75	0.00	98.49	1.83
Bouyant squids	0.01	0.02	0.47	0.38	0.53	99.43	0.95
Pelagic octopods	0.00	0.02	0.21	0.13	0.00	100.00	0.57

**waxing crescent vs first quarter**	**overall = 0.83**						
**species**	**average**	**sd**	**ratio**	**ava**	**avb**	**Cumsum (%)**	**Contribution (%)**
Lanternfishes	0.33	0.30	1.11	9.13	12.38	39.93	39.93
Other invertebrates	0.19	0.25	0.76	10.69	1.12	62.75	22.82
Muscular squids	0.18	0.23	0.78	2.94	4.59	84.27	21.52
Other fish	0.06	0.09	0.63	0.69	0.94	91.38	7.12
Other mesopelagic fish	0.04	0.05	0.78	1.00	0.21	96.33	4.95
Dragonfishes	0.01	0.03	0.39	0.56	0.00	97.59	1.26
Pelagic octopods	0.01	0.03	0.30	0.06	0.12	98.67	1.08
Sepiolids	0.01	0.02	0.37	0.25	0.06	99.72	1.05
Bouyant squids	0.00	0.01	0.17	0.00	0.06	100.00	0.28

**waxing crescent vs full moon**	**overall = 0.80**						
**species**	**average**	**sd**	**ratio**	**ava**	**avb**	**Cumsum (%)**	**Contribution (%)**
Lanternfishes	0.26	0.24	1.07	9.13	12.67	31.87	31.87
Other invertebrates	0.19	0.22	0.86	10.69	7.80	55.57	23.70
Dragonfishes	0.15	0.25	0.61	0.56	19.53	74.71	19.14
Muscular squids	0.10	0.16	0.62	2.94	1.07	87.44	12.72
Other fish	0.05	0.07	0.68	0.69	1.40	93.26	5.83
Other mesopelagic fish	0.04	0.06	0.65	1.00	1.13	98.34	5.08
Sepiolids	0.01	0.02	0.31	0.25	0.00	99.10	0.76
Pelagic octopods	0.00	0.02	0.19	0.06	0.00	99.57	0.47
Bouyant squids	0.00	0.01	0.26	0.00	0.53	100.00	0.43

**first quarter vs full moon**	**overall = 0.86**						
**species**	**average**	**sd**	**ratio**	**ava**	**avb**	**Cumsum (%)**	**Contribution (%)**
Lanternfishes	0.29	0.29	1.02	12.38	12.67	34.12	34.12
Dragonfishes	0.16	0.27	0.61	0.00	19.53	53.08	18.96
Other invertebrates	0.14	0.18	0.79	1.12	7.80	69.49	16.40
Muscular squids	0.11	0.21	0.51	4.59	1.07	82.10	12.62
Other fish	0.10	0.18	0.55	0.94	1.40	93.62	11.51
Other mesopelagic fish	0.04	0.11	0.38	0.21	1.13	98.57	4.95
Bouyant squids	0.01	0.03	0.24	0.06	0.53	99.35	0.79
Pelagic octopods	0.00	0.03	0.17	0.12	0.00	99.90	0.55
Sepiolids	0.00	0.01	0.16	0.06	0.00	100.00	0.10

Table S2. SIMPER of prey groups contributing (%) to dissimilarity among levels of the factor "Lunar irradiance".

**Moderate vs Low**	**overall = 0.79**						
**species**	**average**	**sd**	**ratio**	**ava**	**avb**	**Cumsum (%)**	**Contribution (%)**
Lanternfishes	0.21	0.23	0.92	0.34	2.46	26.69	26.69
Other invertebrates	0.17	0.18	0.92	0.87	1.81	47.73	21.04
Muscular squids	0.17	0.19	0.89	0.90	1.37	68.72	20.99
Other fish	0.11	0.16	0.68	0.63	0.51	82.91	14.19
Other mesopelagic fish	0.05	0.10	0.56	0.23	0.41	89.77	6.86
Sepiolids	0.02	0.05	0.46	0.15	0.21	92.60	2.83
Pelagic octopods	0.02	0.06	0.38	0.11	0.10	95.38	2.79
Bouyant squids	0.02	0.05	0.42	0.15	0.10	97.90	2.52
Dragonfishes	0.02	0.05	0.32	0.00	0.29	100.00	2.10

**Moderate vs High**	**overall = 0.79**						
**species**	**average**	**sd**	**ratio**	**ava**	**avb**	**Cumsum (%)**	**Contribution (%)**
Other invertebrates	0.15	0.17	0.87	0.87	1.74	19.01	19.01
Lanternfishes	0.15	0.18	0.82	0.34	1.95	37.70	18.69
Other fish	0.15	0.19	0.76	0.63	0.84	55.96	18.26
Muscular squids	0.13	0.17	0.79	0.90	0.81	72.67	16.70
Dragonfishes	0.09	0.13	0.67	0.00	1.55	83.53	10.87
Other mesopelagic fish	0.06	0.11	0.56	0.23	0.56	91.46	7.93
Bouyant squids	0.04	0.07	0.55	0.15	0.39	96.25	4.79
Pelagic octopods	0.02	0.06	0.30	0.11	0.03	98.41	2.16
Sepiolids	0.01	0.03	0.37	0.15	0.08	100.00	1.59

**High vs Low**	**overall = 0.76**						
**species**	**average**	**sd**	**ratio**	**ava**	**avb**	**Cumsum (%)**	**Contribution (%)**
Lanternfishes	0.20	0.19	1.03	1.95	2.46	26.42	26.42
Other invertebrates	0.15	0.17	0.92	1.74	1.81	46.51	20.09
Muscular squids	0.13	0.16	0.79	0.81	1.37	63.08	16.57
Other fish	0.10	0.14	0.71	0.84	0.51	75.83	12.75
Dragonfishes	0.08	0.11	0.71	1.55	0.29	86.40	10.57
Other mesopelagic fish	0.05	0.07	0.71	0.56	0.41	93.15	6.74
Bouyant squids	0.02	0.05	0.53	0.39	0.10	96.33	3.18
Sepiolids	0.02	0.04	0.38	0.08	0.21	98.42	2.09
Pelagic octopods	0.01	0.04	0.28	0.03	0.10	100.00	1.58

Table S3. SIMPER of prey groups contributing (%) to dissimilarity among levels of the factor "Current strength".

**Weak vs Intermediate**	**overall = 0.80**						
**species**	**average**	**sd**	**ratio**	**ava**	**avb**	**Cumsum (%)**	**Contribution (%)**
Lanternfishes	0.20	0.27	0.75	0.34	1.94	24.85	24.85
Muscular squids	0.20	0.22	0.91	0.90	1.43	49.53	24.67
Other invertebrates	0.13	0.18	0.73	0.87	1.11	66.19	16.66
Other fish	0.13	0.19	0.67	0.63	0.48	81.90	15.71
Other mesopelagic fish	0.05	0.12	0.44	0.23	0.25	88.45	6.55
Bouyant squids	0.03	0.06	0.43	0.15	0.15	91.90	3.45
Pelagic octopods	0.03	0.07	0.39	0.11	0.12	95.31	3.41
Dragonfishes	0.02	0.06	0.37	0.00	0.41	98.07	2.77
Sepiolids	0.02	0.04	0.39	0.15	0.09	100.00	1.93

**Weak vs Strong**	**overall = 0.79**						
**species**	**average**	**sd**	**ratio**	**ava**	**avb**	**Cumsum (%)**	**Contribution (%)**
Lanternfishes	0.18	0.17	1.03	0.34	2.43	22.54	22.54
Other invertebrates	0.18	0.17	1.01	0.87	2.18	44.86	22.32
Muscular squids	0.13	0.15	0.84	0.90	0.97	60.84	15.98
Other fish	0.13	0.17	0.74	0.63	0.75	76.80	15.96
Other mesopelagic fish	0.06	0.09	0.66	0.23	0.60	84.54	7.74
Dragonfishes	0.06	0.11	0.52	0.00	1.04	91.99	7.45
Bouyant squids	0.03	0.05	0.50	0.15	0.26	95.44	3.45
Sepiolids	0.02	0.05	0.43	0.15	0.20	97.99	2.55
Pelagic octopods	0.02	0.05	0.32	0.11	0.04	100.00	2.01

**Strong vs Intermediate**	**overall = 0.77**						
**species**	**average**	**sd**	**ratio**	**ava**	**avb**	**Cumsum (%)**	**Contribution (%)**
Lanternfishes	0.21	0.20	1.05	2.43	1.94	27.09	27.09
Other invertebrates	0.16	0.17	0.92	2.18	1.11	47.46	20.37
Muscular squids	0.14	0.16	0.85	0.97	1.43	65.46	18.00
Other fish	0.10	0.15	0.67	0.75	0.48	78.24	12.78
Dragonfishes	0.07	0.11	0.61	1.04	0.41	86.73	8.49
Other mesopelagic fish	0.05	0.07	0.70	0.60	0.25	93.29	6.57
Bouyant squids	0.02	0.04	0.48	0.26	0.15	96.02	2.73
Sepiolids	0.02	0.04	0.38	0.20	0.09	98.15	2.12
Pelagic octopods	0.01	0.04	0.32	0.04	0.12	100.00	1.85
